# Deep sexual dimorphism in adult medaka fish liver highlighted by multi-omic approach

**DOI:** 10.1038/srep32459

**Published:** 2016-08-26

**Authors:** Qin Qiao, Séverine Le Manach, Benoit Sotton, Hélène Huet, Evelyne Duvernois-Berthet, Alain Paris, Charlotte Duval, Loïc Ponger, Arul Marie, Alain Blond, Lucrèce Mathéron, Joelle Vinh, Gérard Bolbach, Chakib Djediat, Cécile Bernard, Marc Edery, Benjamin Marie

**Affiliations:** 1 UMR 7245 MNHN/CNRS Molécules de Communication et Adaptation des Micro-organismes, Sorbonne Universités, Muséum National d’Histoire Naturelle, Paris, France; 2 Université Paris-Est, Ecole Nationale Vétérinaire d’Alfort, BioPôle Alfort, Maisons-Alfort, France; 3 UMR 7221 CNRS/MNHN, Évolution des Régulations Endocriniennes, Sorbonne Universités, Muséum Nationale d’Histoire Naturelle, Paris, France; 4 UMR 7196 MNHN/CNRS, INSERM U1154, Sorbonne Universités, Museum National d’Histoire Naturelle, Paris, France; 5 Institut de Biologie Paris Seine/FR 3631, Plateforme Spectrométrie de masse et Protéomique, Institut de Biologie Intégrative IFR 83, Sorbonne Universités, Université Pierre et Marie Curie, Paris, France; 6 USR 3149 ESPCI/CNRS SMPB, Laboratory of Biological Mass Spectrometry and Proteomics, ESPCI Paris, PSL Research University, Paris, France

## Abstract

Sexual dimorphism describes the features that discriminate between the two sexes at various biological levels. Especially, during the reproductive phase, the liver is one of the most sexually dimorphic organs, because of different metabolic demands between the two sexes. The liver is a key organ that plays fundamental roles in various physiological processes, including digestion, energetic metabolism, xenobiotic detoxification, biosynthesis of serum proteins, and also in endocrine or immune response. The sex-dimorphism of the liver is particularly obvious in oviparous animals, as the female liver is the main organ for the synthesis of oocyte constituents. In this work, we are interested in identifying molecular sexual dimorphism in the liver of adult medaka fish and their sex-variation in response to hepatotoxic exposures. By developing an integrative approach combining histology and different high-throughput omic investigations (metabolomics, proteomics and transcriptomics), we were able to globally depict the strong sexual dimorphism that concerns various cellular and molecular processes of hepatocytes comprising protein synthesis, amino acid, lipid and polysaccharide metabolism, along with steroidogenesis and detoxification. The results of this work imply noticeable repercussions on the biology of oviparous organisms environmentally exposed to chemical or toxin issues.

Sexual dimorphism terminology is widely used for organisms that perform sexual reproduction to describe physiological differences between two sexes at various biological levels. Although sexual dimorphism is generally considered at the anatomical or the behavioural level, it can also be extended to differences in the physiology of functions not directly involved in reproductive processes. According to different strategies for survival fitness of the two sexes and the consideration of their respective sexual specificities, exogenous perturbations could induce noticeable dissimilarities of endogenous response of individuals of the two sexes. Recent research observations have shown that a large number of genes exhibited deep sexual differences at the transcriptomic level in various tissues, suggesting that, in fact, sex-dependent genetic and hormonal regulations could also affect non-gonadal organs such as brain or liver^1^,^2^,^3^. This assumption is supported by numerous reports that have addressed that males and females may differ in their susceptibility to environmental or biological stresses, as well as in the differential responsiveness of the liver to various xenobiotics^4^.

The liver is a key organ that plays fundamental roles in various physiological processes, including digestion, energetic metabolism, xenobiotic detoxification, biosynthesis of serum proteins, and also in endocrine or immune responses. Because of the different metabolic needs between sexes, especially during the reproductive phase, the liver is one of the most sexually dimorphic organs in terms of gene expression^5^. The first evidence of a sex-related difference in the rat hepatic steroid metabolism was published in 1953^6^. Based on these initial observations, five decades of research have since then established the existence of a gonadal-hypothalamo-pituitary-liver axis that determines the differences between male and female liver. Moreover, the importance of hormone secretion patterns has been revealed and the understanding of hepatic gene regulation at the molecular level has advanced in mammals^7^. For example, various studies have shown that many hepatic genes associated with xenobiotic metabolisms, such as cytochrome P450, are expressed in a sex-dependent manner during the detoxification process^4^. Particularly, the sex-dimorphism of the liver is obvious in oviparous animals, as the female liver is the main organ for the synthesis of oocyte constituents, such as the yolk protein precursors (vitellogenins) and the zona pellucida proteins (choriogenins)^8^.

As continental aquatic environments are threatened by a large spectrum of xenobiotics and pollutants, freshwater oviparous organisms such as fish are especially impacted by these potential toxicants, and their liver detoxification capabilities constitute essential defences for the fitness of these organisms. In this context, one can suppose that the various sexual dimorphisms of oviparous organisms, concerning energetic metabolism, detoxification and reproduction processes may drastically influence the hepatic responses of different sexes.

In this work, we were interested in identifying the molecular sexual dimorphism in the liver of adult medaka fish and illustrating its implication in response to hepatotoxic exposures. Small fish such as the Japanese medaka (*Oryzias latipes*) have emerged as useful vertebrate model organisms, suitable for studying various physiological processes^9^, toxicological mechanisms^10^ and also ecotoxicological effects^11^. Medaka fish presents the advantages of small size, established models produced from inbred lines, rapid development and growth, high fecundity, omnivorousness, and also shows sugar and lipid metabolic profiles similar to those of mammals^9^. By developing an integrative approach comprising histology and different high-throughput omic investigations (*i.e.* metabolomics, proteomics and transcriptomics), we are now able to globally describe the sex-dimorphism in the medaka liver. To our knowledge, this constitutes the first systematic investigation of the liver sex-dimorphism in this model organism. Furthermore, under hepatotoxin perturbed conditions, sex-specific variation in molecular responses was investigated using quantitative proteomic analyses, implying potential different repercussions on the biology of fish environmentally exposed to chemical issues.

## Results

### Histology

**Figure 1:**
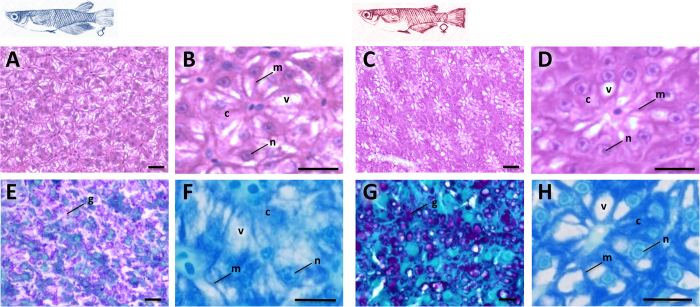
Histological investigations of male and female medaka livers. Representative histological observations under a light microscope of thick or thin sections of adult medaka liver stained with HES (**A**–**D**), PAS (**E**,**G**), and toluidine blue (**F**,**H**), for males (**A**,**B**,**E**,**F**) and females (**C**,**D**,**G**,**H**). Scale bares represent 20 μm. g, glycogen reserve; n, nucleus; m, membrane; c, cytosol; v, vesicle.

The liver of organisms under undisturbed lab condition presents in both sexes a characteristic architectural organization with polyhedral hepatocytes organized around the capillary sinusoids and the bile canaliculi, appearing in characteristic cord-like parenchymal structures. As shown in Fig. 1, medaka fish liver presents a sexual dimorphism at the cellular level based on histological observations of the hepatocytes. Indeed, male and female hepatocytes present obvious differences in their global cytoplasm appearance with a distinct distribution of vesicles that are revealed using hematoxylin-eosin-saffron (HES), periodic acid-Schiff/alcian blue (PAS) or toluidin blue staining. Whereas female hepatocytes present large isolated reserve vesicles (mostly one per hepatocyte) with dense contents of glycoprotein and/or glycogen (Fig. 1C,D,G,H), male hepatocytes exhibit more diffuse small vesicles (Fig. 1A,B,E,F).

### NMR metabolomics

**Figure 2:**
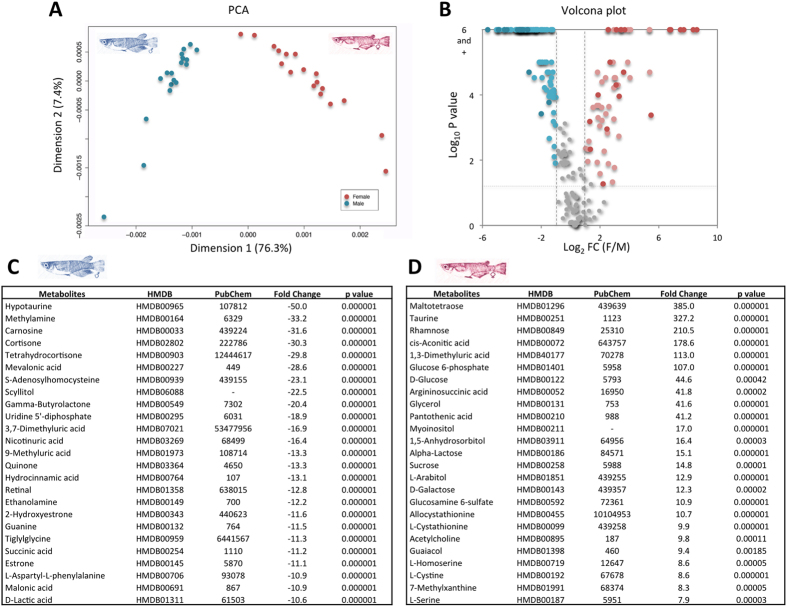
Metabolomics of male and female medaka livers by ^1^H NMR. Principal component analysis (PCA) performed with the quantification values of the 237 metabolites detected by Batman’ R package algorithm (Supplementary Table S2) from the 18 males and 18 female individual NMR spectra (**A**). Volcano plot representation of the 237 metabolites according to female/male fold change average and significance of the differences (|log_2_ FC| > 1 corresponding to |FC| > 2 and log_10_ Pvalue < 1.3 corresponding to Pvalue < 0.05) (**B**). Female and male over-represented metabolites are determined with positive and negative significant FC (F/M) values and are shown in red and blue, respectively, and are represented with darker colours when metabolite presenting VIP values are superior to 1, according to PLS-DA analysis. Top-25 lists of the putative annotations of male- and female-representative metabolites (**C** and **D**, respectively).

The hydrophilic fraction of the liver of medaka bred under undisturbed lab condition was investigated on 18 males and 18 females by ^1^H NMR analysis as shown in Fig. 2. Up to 237 different potential metabolites have been detected and relatively quantified according to the Batman R package analysis. The global analysis of the molecular pathway involved in liver metabolism reveals that the medaka liver metabolome presents a significant enrichment in a very wide diversity of processes, comprising principally glutathione, taurine, amino acid, carbohydrate, lipid, steroid hormone and tricarboxylic acid (TCA) cycle metabolisms (supplementary Table S1). Although our metabolomic analysis was performed on the hydrophilic fraction of the liver, we were able to observe various mostly hydrophobic metabolites, such as steroidal compounds, that testify of the intense lipid metabolism, especially in males. Principal component analysis (PCA) clearly discriminates specific liver metabolome between males and females according to its component 1 that comprises 76% of the total sample variation (Fig. 2A).

Significantly over-represented metabolites in both sexes (|FC| > 2 and Pvalue < 0.05) were highlighted in a volcano plot (Fig. 2B). Whereas 59 molecules are over-represented in females, 103 appear to be over-represented in male metabolome (supplementary Table S2). According to their putative annotation provided by the Batman algorithm, a pathway enrichment analysis indicates that female-enriched metabolomes would rather be implicated in some saccharide and amino acid metabolic processes, whereas male-enriched metabolomes seem to exhibit signatures of steroid hormone biosynthesis and energy (TCA, nitrogen) processes, when the common metabolite set is more relevant to other amino acid and saccharide metabolism (Fig. 2C,D). Another interesting noticeable specificity of the medaka fish metabolome concerns the relative quantity of taurine and hypotaurine that are strongly over-represented in females and males, with 327 and 50 |FC|, respectively.

### Proteomics

The cytosolic fraction of the liver proteome of medaka bred under undisturbed lab condition was investigated by high-throughput method of bottom-up proteomics. The trypsin-cleaved peptides were separated by nano-LC and analysed by high-resolution mass spectrometry, then the proteins were identified thanks to a large genomic dataset available for the Japanese medaka^12^. Among the 820 proteins identified in the three male pools, 64 appear to be potentially male-biased, because they were detected in both 3 male pools, and not in any of the three female pools. For females, 178 seem to be similarly biased of the 934 total proteins identified in the three female pools (Fig. 2A), and not in any male pool. This large protein sex-biased composition of the liver is also illustrated with semi-quantification of the proteins according to both MS and MS/MS data, represented in a volcano plot (Fig. 2B). Among 1241 identified proteins, 25 and 125 are significantly over-represented in males and females, respectively (|FC| > 3 and p < 0.01) (Supplementary Table S3). A global analysis of these sex-biased proteins according to the described functions of their human orthologs suggests that female-biased proteins are significantly related to tRNA and nucleotide sugar metabolism, that might be related with intense gene expression and synthesis processes, whereas male-biased proteins are related to bile and amino acid metabolisms, and common proteins to other TCA, sugar, lipid and amino acid metabolisms, characteristic of classical liver metabolic pathways (Supplementary Table S3).

**Figure 3:**
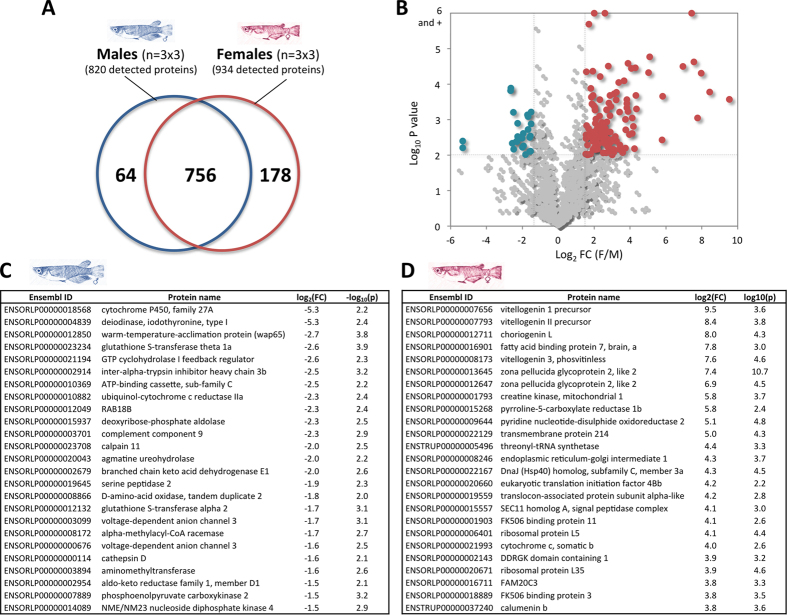
Proteomics of male and female medaka livers. Unscaled Venn’s diagram of the protein identified with at least 95% protein identification certainty in all of the 3 different 3-individual pools of male and/or female medaka livers (**A**). Volcano plot representation of the 1241 proteins according to female/male fold change average and significance of the differences (|log_2_ FC| > 1.5 corresponding to |FC| > 3 and log_10_ Pvalue < 2 corresponding to Pvalue < 0.01) determined according to Scaffold 4.5.1 semi-quantitative values based on both MS and MS/MS data (**B**). Female and male over-represented proteins are determined with positive and negative significant FC (F/M) values and are shown in red and blue, respectively. Top-25 lists of the male- and female-superabundant proteins (**C** and **D**, respectively).

Among the top highly-abundant proteins in female livers dominate various isoforms of vitellogenins and choriogenins, together with fatty acid-biding, cytochrome P450 and various isoforms of ribosomal and translation-related proteins, whereas male-enriched liver proteins interestingly present other cytochrome P450 (CYP) isoforms, complement proteins, glutathione S-transferases (GSTs) and various TCA metabolism-related proteins, together with wap65 - a protein of unknown function whose transcript over-expression appears characteristic of the male Gulf pipe *Syngnathus scovelli*^13^ - highlighting important singularities of the liver proteome in both sexes (Fig. 3C,D).

### Transcriptomics

**Figure 4:**
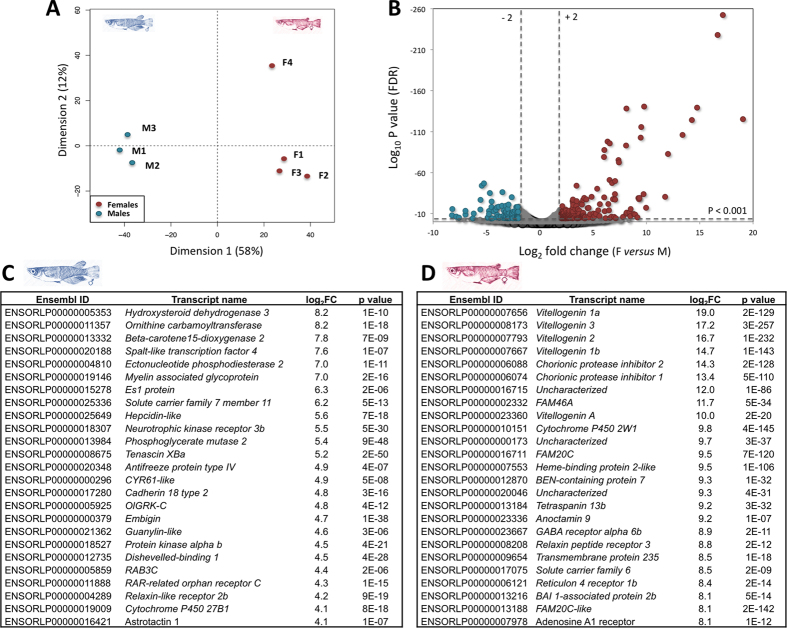
Transcriptomics of male and female medaka livers investigated by RNA-seq approach. Principal component analysis (PCA) performed according to the transcript count for the 16,523 genes encoding medaka liver proteins (Supplementary Table S4) from the 3 male and 4 female pooled cDNA sequenced by Hiseq 1000 comprising at least 30 million reads per libraries (**A**). Volcano plot representation of the gene expression according to female/male fold change average and significance of the differences (|log_2_ FC| > 2 corresponds to |FC| > 4 and log_10_ Pvalue < 3 corresponds to Pvalue < 0.001) (**B**). Female and male over-expressed genes are determined with positive and negative significant FC (F/M) values and are shown in red and blue, respectively. Top-25 lists of the male- and female-over-expressed genes (**C** and **D**, respectively).

PCA clearly discriminates between all male and female liver transcriptomes of medaka bred under undisturbed lab condition investigated by RNA-Seq analysis (Fig. 4A), and the volcano plot representation indicates a colossal over-expression of some genes in females compared with males (Fig. 4B). Indeed, some genes such as *vitellogenin 1*, *3* or *6* reach above 15 to 19 |log_2_ FC| variations (namely up to 500,000 |FC|) (Supplementary Table S4), representing a large portion of the total female liver transcriptome. In contrast, in male livers the most over-expressed gene, *hydroxysteroid dehydrogenase 3*, exhibits only up to 8 |log_2_ FC| (≈250 |FC|) variation in comparison with females. Additional pathway analyses, performed with the 375 and 147 significantly enriched transcripts in females and males, considering their human orthologs (Supplementary Table S4), indicate global enrichments (p < 0.1) of steroid and amino acid processes in both sexes when males also exhibit terpenoid-quinone and fatty acid biosynthesis over-representations.

The list of the over-expressed genes in females comprises, along with some genes whose expressions are well known to be female-specific, such as *vitellogenins*, *choriogenins* and *chorionic protease inhibitors*, various forms of *FAM20C* genes, belonging to the serine threonine kinase 20c-like family. Various isoforms of the FAM20C protein family were similarly over-expressed in the female liver of the Gulf pipefish^13^. Although the biological function of the proteins of this family remains poorly documented, they are interestingly annotated in GO library as “cellular response to estrogen stimulus” genes, and may constitute female-specific markers in fish livers. With some agreement with our proteomic observation, other genes of interest belong to the *cytochrome-P450* (*CYP*) family. Some member such as, for example, *cytochrome-P450 27b1* and *cytochrome-P450 2w1* appear clearly over-expressed in male and female medaka livers, respectively (Fig. 4C,D).

### Integrated pathway analysis

**Figure 5:**
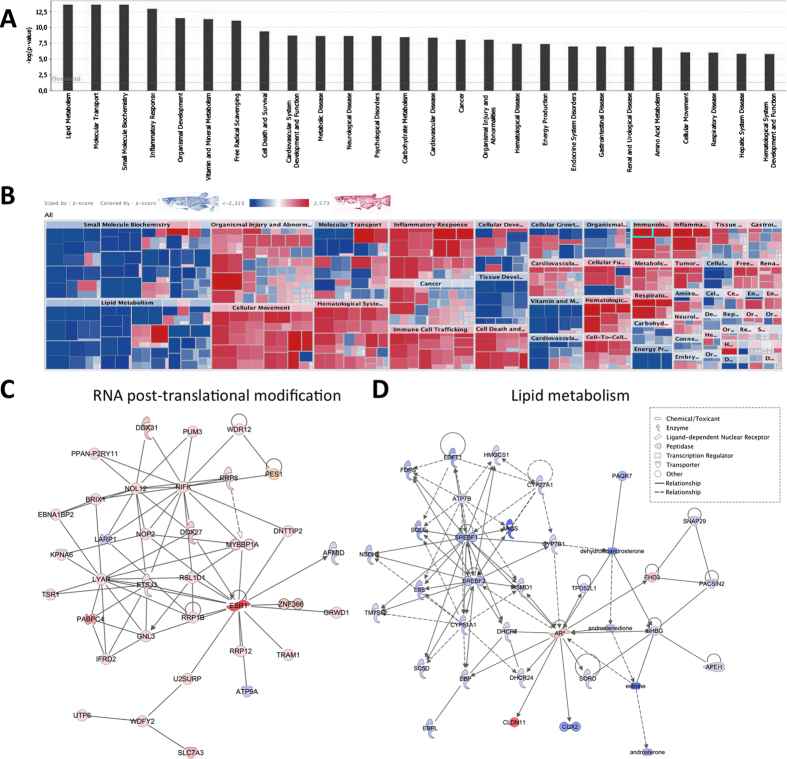
Ingenuity pathway analysis performed on male *versus* female fold change values (M/F FC) of both transcriptomic and metabolomic data. Top dysregulated molecular pathways represented on bar chart (**A**) and heatmap (**B**). Molecular functions that are specifically activated in males and females (Supplementary Table S6) are indicated in blue and red, respectively. Examples of significant molecular networks related to RNA post-translational modification (**C**) and lipid metabolism (**D**) processes (score = 38 and 28). Relative up-regulation of transcripts and metabolites in males or in females are indicated in blue and red, respectively.

To investigate and visualize the biological connectivity of the sex-enriched metabolites and transcripts, the network-generating algorithm of ingenuity pathway analysis (IPA) was used to maximize the interconnectedness of molecules based on all known connectivity in the database developed from Human molecular knowledge in the liver. The results of the IPA biological function analysis (Supplementary Table S6), represented as a bar chart and a heatmap, are shown in Fig. 5A,B, respectively. Lipid metabolism, molecular transport, small molecule biochemistry, inflammatory response, organismal development, vitamin and mineral metabolism, and free radical scavenging appear to be the most significantly represented functional categories according to the combined liver transcriptome and metabolome dataset. The sex-specificity of the molecules involved in these processes is indicated in the heatmap representation (Fig. 5B) that clearly shows a global up-regulation of molecules involved in small molecule biochemistry, lipid metabolism, tissue development, vitamin and mineral metabolism, energy production and carbohydrate metabolism in males, whereas in females most of the molecules involved in cellular movement, haematological system, inflammatory response, or immune cell trafficking appear largely up-regulated.

The IPA network search shows that 2 of the top networks consisted predominantly of only female- and male-enriched molecules are related respectively to RNA post-transcriptional modification and lipid metabolism processes, as shown in Fig. 5C,D. These molecular network representations clearly illustrate the selected massive induction of some genes and metabolites related to RNA post-translational modification in females (Fig. 5C), and specific lipid metabolism processes connected with cholesterol metabolism and steroidogenesis, in males (Fig. 5D).

### Specificities of the toxicological molecular responses

**Figure 6:**
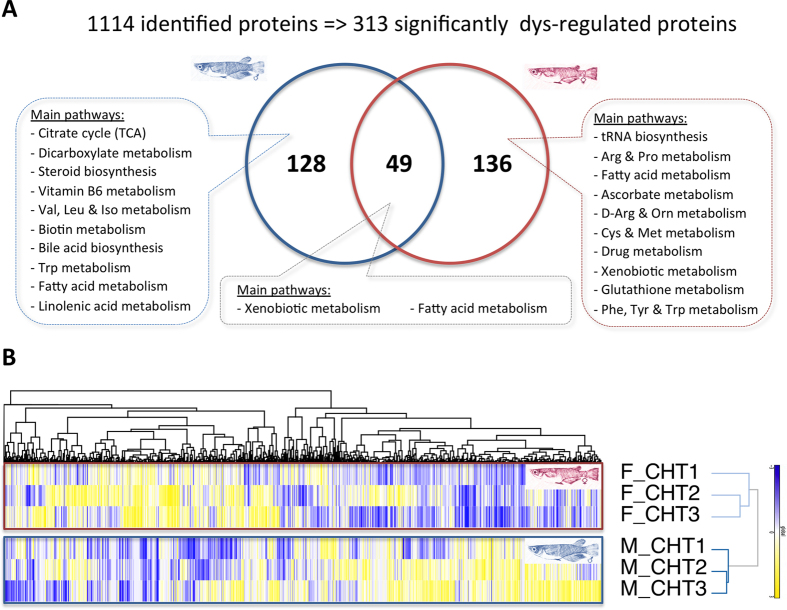
Global proteomic effects of the different hepatotoxic cyanobacterial extract exposures (CHT1-3) on medaka fish liver. Venn’s diagram (**A**) and heatmap representation (**B**) of proteinaceous dysregulation investigated by iTRAQ quantitative proteomic approach for both males and females. Samples are normalized according to male and female controls and significance of protein dysregulation was settled at |log_2_FC| > 0.25. Main metabolic categories of dysregulated proteins in males, females or in both sexes were determined according to Metaboanalyst 3.0 molecular pathway searches (Supplementary Table S5). Down-regulated proteins are indicated in yellow, up-regulated proteins in blue, and missing values in grey. Clustering was performed according to Pearson’s correlation coefficient values.

The quantitative proteomic analysis of liver of the medaka exposed to various hepatotoxic treatments was performed on nanoLC-ESI-MS/MS, leading to the identification of 1114 proteins and to the differential quantification of 177 and 185 proteins in males and females, respectively (Fig. 6A). Only above a quarter of them (49 proteins) appears to be common between the two sexes. Protein quantification in each group of adult medaka fish, chronically exposed to the various cyanobacterial hepatotoxic treatments (CHT1-3) was reported, according to the relative intensity normalized with the controls set at 0, and the reliable quantifications in male and female livers were represented in a heatmap with hierarchical cluster analysis. This analysis clearly revealed a distinguishable sex-dependent response of medaka fish to the various cyanobacterial hepatotoxic treatments according to the group distribution given by the clustering analysis, which is based on the pattern of relative abundance of up- and down-regulated proteins (Fig. 6B). These cyanobacterial hepatotoxic treatments dysregulate various proteins of various molecular function categories comprising lipid, amino acid, carbohydrate and TCA metabolisms and detoxification processes (Supplementary Table S5). The sex-specificity of the liver molecular response suggests that identical hepatic stress could impact these various molecular processes differently, potentially inducing dissimilar biological repercussions in the organisms of two sexes. In our example, male-enriched protein dysregulations concern rather TCA, steroid, fatty acid, amino acid and vitamin B6-7 metabolism pathway categories, whereas female-enriched protein dysregulations are rather related to tRNA biosynthesis, amino acid, glutathione, xenobiotic and drug metabolism pathways.

## Discussions

The liver is a key organ in vertebrates performing a large diversity of vital functions, including processing and storage of nutrients, maintenance of serum composition, bile production, and xenobiotic detoxification. It is primarily an exocrine gland, secreting bile into the intestine, but it is also an endocrine organ and a blood filter. The liver is a metabolic factory, which synthesizes and breaks down a variety of substances, comprising the production of bile salt anions, the synthesis of urea and many plasma proteins, the metabolism of glycogen, cholesterol and fatty acids, the detoxification of many drugs, and the processing of steroid hormones and vitamin D. Studies carried out in rodents have established that sex-based differences in liver function also characterize many drug-metabolizing enzymes (DMEs), including sulfotransferases, glutathione S-transferases, P450s and other steroid metabolizing enzymes^4^. The sexual dimorphism of liver gene expression is not confined to DMEs, and it concerns more than 1000 genes in these organisms^2^, including plasma lipoproteins, pheromone binding proteins, regulators of fatty acid homeostasis, nuclear receptors, and other transcription factors^3^.

Similar to the mammalian liver, the teleost liver plays an important role in the metabolic homeostasis of the whole organism, in addition to that, oviparous vertebrate-specific processes related to the synthesis of various oocyte protein precursors (*i.e.* mainly vitellogenins and choriogenins, together with other minor vitamin-binding proteins) are synthesized in females under the direct control of estrogens, which bind to estrogen receptor complex and activate the translation of messenger RNA via *cis*-regulation mechanisms^8^,^14^. The massive rate of synthesis of vitellogenin in the egg-laying animal causes considerable ultrastructural changes in liver cells, which are characterized by extensive proliferation of the rough endoplasmic reticulum and the Golgi apparatus^15^. In mature female medaka, a remarkable part of the liver metabolism might be dedicated to these reproduction-related processes, as each female can spawn above 30 mature oocytes daily^16^. This massive synthesis is known to induce large cellular and molecular modifications, as it can also increase the lipid synthesis of hepatocytes^5^. This metabolic adjustment to maintain the reproductive competency of the female constitutes one of the physiological bases for the extended sexual dimorphism in fish livers.

Our histological observations of male and female medaka livers clearly showed differences of reserve vesicle within hepatocytes, which is consistent with ultrastructural modifications in the liver cells between males and females. Interestingly, previous observation of mature medaka liver under transmission electron microscope shows that, in the perinuclear region, granular endoplasmic reticulum, mitochondria, and peroxisomes appear largely increased in number in female hepatocytes^15^. These hepatocyte sexual differences in cellular organization and content might be related to the intense activity of protein synthesis and consequently the high energy requirement of female hepatocytes. Indeed, in sexually mature fish, as in other oviparous vertebrates, livers globally present morphological, molecular and functional sexual-dimorphisms^15^,^17^,^18^. The liver of female performs an important function in the synthesis of a large set of proteins involved in the oocyte envelop and vitellogen reserves, whereas male liver hepatocytes do not exhibit such activity.

Although the precise function of taurine and hypotaurine, which were highly abundant in female and male livers, respectively, and the balance between the two remains poorly documented in fish liver^19^, one previous study has reported the influence of taurine on egg maturation^20^. To date, only one investigation performed in adult zebrafish has attempted to compare male and female metabolomes, according to various analytical approaches, including GC-MS, LC-MS and NMR, and has observed a significant up-regulation in various fatty acids, together with valine, acetate, glutamate, glutamine, creatinine and betaine in female liver^21^.

The sex-biased pattern depicted in our transcriptomic analyses appears acutely congruent and even more contrasted than our global proteome investigation. These strong sex-biases testify of the intensity of the female liver efforts for the gene expression and the synthesis of the oocyte precursor proteins^8^,^14^, and the involvement of the male liver in steroid hormone and metabolism processes^22^, such as urea and energy cycles^9^, respectively.

Previous investigation of medaka fish has observed that liver transcriptome globally exhibits significant enrichment in the expression of genes related to macromolecule, RNA, and nitrogen compound metabolic processes with regard to the gene expression in other tissues, but it has not considered the differences between sexes^23^,^24^. Previous works performed on zebrafish and Gulf pipefish have highlighted large sets of genes whose expression appears to be driven by sex-dependent processes^16^,^25^. On one side, zebrafish transcriptomic approach reveals that the female-over-expressed gene list included *vitellogenins* and *zona pellucida glycoproteins*, many ribosomal proteins, and *estrogen receptor 1*, in contrast, the list of male-over-expressed genes contains *fatty acid-binding protein2*, *apolipoprotein 4*, and also genes that are supposed to be involved in anti-inflammatory processes, such as *complement factors 9b* and *3b*, together with several *chitinases*^25^. On the other side, transcriptomic investigation performed on Gulf pipefish shows a quite similarly high over-expression in characteristic genes of females, such as *vitellogenin b* and *c*, *choriogenin h*, and *zpc 4-like*, in addition to *estrogen receptor 1* and various *fam20c* isoforms, whereas males exhibit less intense over-expression of specific genes, comprising various metabolism related genes such as *hint3*, *ctl*, *nsun3* and *wap65*^13^. Moreover, primary investigation of the medaka fish liver transcriptome by microarray analysis indicates that the female-specific transcript list comprises some previously characterized female-specific genes such as *vitellogenins*, *choriogenins*, *ZP* family genes, *cyclins B* and *42S nucleoprotein*, whereas most of the male-specific transcripts have not yet been assigned or characterized^26^.

Similarly, previous proteinaceous investigations have highlighted that female liver proteome moreover contains massive amounts of oocyte precursor proteins (*i.e.* vitellogenins, choriogenins and fatty acid-binding proteins) that are being secreted by the hepatocytes^17^, together with variations between sexes in drug metabolism capabilities^4^. CYPs constitute a diverse group of enzymes that are potentially involved in key reactions of oxidation of organic substances, such as drug detoxification^17^ and steroid hormone metabolism^4^,^7^,^13^. The sexual polymorphism in the expression of these enzymes may have fundamental repercussion on liver physiology such as drug-metabolism processes.

In addition to our transcriptomic data that gives a congruent view with previously published observations, our systematic investigation constitutes an unprecedented opportunity to globally depict the medaka liver sexual dimorphism at different molecular and cellular levels. By developing an integrative approach combining high output proteomic, RNA-Seq transcriptomic and non-targeted metabolomics outputs, together with histological examinations, we are able to appreciate the wideness and the deepness of the sexual dimorphism, in terms of both number and intensity of the sex-dependent dysregulations. At the mRNA level, twice more sex-over-expressed transcripts appear in females, comprising some genes involved in ovogenesis (*i.e. vitellogins* and *choriogenins*), and reach very high fold change values (up to 50,000 and 250 FC in females and males, respectively). Proteomic investigations also show more proteins that appear to be specific of female proteomes, with higher fold change too (up to 750 and 50 FC in females and males, respectively). The quantitative metabolome analysis performed by NMR indicates that the difference of fold changes between female- and male-enriched metabolites appear to be much higher in female (up to 350 and 50 FC in females and males, respectively). The observations of globally more intensive and numerous molecular up-regulations in female livers are in agreement with the conception of an oviparous female liver that is in charge of extra metabolic activity, according to their massive production of oocyte yolk stocks and chorion precursors, which have substantial impacts on both amino acid, saccharide and fatty acid metabolism of the global liver activities^8^.

As the mature female liver is considered to be more physiologically and energetically solicited than male’s, we assume that female liver could be consequently more susceptible to hepatotoxic stressors. In our experiments and according to other investigations on small fish ecotoxicology, various hepatotoxic stressors such as cyanotoxins^11^, pesticides^27^,^28^ or aromatic hydrocarbons^29^ induce a higher toxicological response in female livers, suggesting that females would be more sensitive to the effects of those molecules than males^15^,^17^,^18^. However, some examples also attest to a higher susceptibility of male livers according to certain specific exposure conditions to toxic chemicals^30^, and a careful investigation of the dimorphic detoxification capabilities should aim at being performed for each specific chemical or hepatic stressor evaluation. Indeed, as the liver, being the principal detoxification organ, presents noticeable sex-differences in its drug metabolism (*e.g.* CYP P450 isoforms) and homeostasis capabilities^4^, it is likely that for some hepatic stressors the detoxification performance, which could be clearly distinguishable between the two sexes, should be systematically considered in environmental toxicology evaluations.

Overall, in addition to providing first insight into the molecular mechanism underlying the sex-specificity of the livers of oviparous organisms, and concerning important liver processes, such as energetic metabolism, detoxification and reproduction, our molecular integrated research demonstrates also that, freshwater oviparous organisms such as medaka fish present a net sex-dimorphism in the molecular response specifically induced by chronic hepatotoxin exposures.

Furthermore, numerous reports show that fish populations are adversely affected by environmental estrogens, which can potentially present ecological adverse effects, via the induction of the synthesis of oogenesis proteins. These xenoestrogens can also impact various liver processes that also exhibit sex-dimorphisms, such as metabolism and biotransformation enzymes that directly influence the stress resistance capabilities of the organism. Additional studies are still needed to validate these findings at higher levels of biological organization, and to fully estimate their consequences for different populations of the global ecosystem.

## Methods

### Medaka fish

Medaka fish (*Oryzias latipes*) of the inbred Cab strain were used for this experiment. The animals were handled and experiments were performed in accordance with European Union regulations concerning the protection of experimental animals and the experimental procedures were approved (N°68-040 for 2013-18) by the “Cuvier’s ethical committee” of the Muséum National d’Histoire Naturelle (French national number C2EA - 68). All fish used in this study (during summer 2014) originate from the same broodstock (F0 from genitors provided in November 2013 by the Amagen CNRS/INRA platform - Gif-sur-Yvette, France).

All histopathology, metabolomics, proteomic and transcriptomic analyses, except for iTRAQ proteomic with chronically exposed fish, were performed on the untreated fish breed and kept under control conditions, described as follow. Six month-old adult fish (around 0.55 ± 0.08 and 0.59 ± 0.10 g, for males and females, respectively; n = 36), mature and sexually active (with secondary sexual character well developed; sex determination was confirmed by further histology of the gonads for 36 individuals), were maintained at 25 ± 1 °C, with 15 h:9 h light/dark cycle (in reproductive cycle). Fish were raised in 20 L glass aquaria (in triplicate tanks, containing above 10 male and 10 female per tank) filled with a continuously-aerated mixture of tap water and reverse osmosis filtered water (1/3–2/3, respectively), which was renewed once a week. Fish were fed three times a day with commercial food for juvenile salmon, supplemented once a day with fresh artemia, and were inspected three times daily, and no abnormal behaviour, nor mortality was observed. Fish were randomly selected, briefly anesthetized in buffered 0.1% MS-222, sacrificed. The liver samples were collected and prepared for further analysis, as described below.

Mature and sexually active fish, bred in the same conditions of light, temperature and nourishing, were used for chronic exposure to hepatotoxin then for iTRAQ quantitative proteomic analysis, as further described in the following paragraph. Five month-old fish were transferred to experimental tanks (15-L glass tanks, containing 5 males and 5 females each) two weeks prior to the beginning of the experimentation.

### Hepatotoxic mixture exposure

Fish were exposed during 21 days to environmental concentrations of 3 different cyanobacterial hepatotoxic treatments^31^ (CHT), called CHT1-3, as well as to solvent control conditions (Control). The experiment was performed in triplicate tanks for each treatment (comprising 30 fish, 15 males and 15 females per treatment) and the exposure conditions were maintained by the renewal of two-thirds of the total aquaria volume (10 L) containing hepatotoxic mixture every 2 days. Fish were inspected three times daily, and no abnormal behaviour, nor mortality was observed during all the experimentation. At the end of the experiment, fish were anesthetised in 0.1% MS-222, euthanized and then livers were sampled for further analyses using iTRAQ quantitative proteomics.

### Histopathology

Liver samples from at least 9 males and 9 females bred under unstressed control conditions were fixed in cold 10% buffered formalin (4 °C, 48 h), then transferred into 70% ethanol, dehydrated in successive baths of ethanol (from 70 to 95%), and then embedded in paraffin. Blocks were cut in 3–5 μm-thick sections, and slides were stained with hematoxylin-eosin-saffron (HES) or periodic acid-Schiff/alcian blue (PAS), according to the standard histological procedure. Alternatively, liver samples were fixed with a mixture of paraformaldehyde (2%), glutaraldehyde (0.5%), picric acid (0.5%) and sucrose (0.18 M) in 0.1 M pH 7.4 Sørensen buffer prior to post-fixation in osmium tetroxide (1%). Samples were then dehydrated in ethanol, embedded in the epoxy mixture (Spurr’s resin), and cut in semi-thin 0.5 μm-thick sections and stained with toluidine blue (TB).

### Metabolome ^1^H-NMR spectra

Liver extraction was carried out using the methanol/chloroform/water method (ratio 2/2/1.8). Fresh frozen livers of 18 individuals for each sex (6 individuals from each triplicate tank) bred under unstressed control conditions were weighted and then homogenized in the ice cold methanol (8 mL.g^−1^ of tissue; AnalaR Normapur, min. 99.8%) and ice cold milliQ water (2.5 mL.g^−1^) and then vortexed for 1 min. Subsequently, ice cold chloroform (4 mL.g^−1^; Normapur, 99.3%) and milliQ water (4 mL.g^−1^) were added. Then, the mixture was vortexed for 1 min and incubated on ice for 10 min to partition. The supernatant was then centrifuged at 4 °C for 10 min at 2,000 g. The upper polar fraction was then transferred to 2 mL Eppendorf tubes, dried under Speed-vac device and then kept at −80 °C until NMR analysis. The extracts were dissolved in 550 μL of 0.1 M sodium phosphate buffer prepared in D_2_O (10% v/v) containing 0.25 mM sodium-3-tri-methylsilylpropionate (TMSP) as an internal standard, then were transferred to a 5-mm NMR tube (Norell, France) and analyzed immediately by ^1^H-NMR.

All NMR data were recorded at 298 K on a 600 MHz Bruker AVANCE III HD spectrometer equipped with a 5 mm TCI CryoProbe (^1^H-^13^C-^15^N) with Z-gradient. One-dimensional ^1^H NMR spectra were acquired using a standard Bruker noesygppr1d pulse sequence to suppress water resonance. Each spectrum consisted of 512 scans of 32,768 data points with a spectra width of 7.2 kHz, a relaxation delay of 3 s and an acquisition time of 2.3 s. A Quality Check (QC) sample was injected every 6 samples, in order to verify that no significant drift of the analysis occurs, according to expected reference. Spectra were then processed with Topspin software (Bruker) for alignment and noise reduction, and analyzed for bucketing, annotation and quantification with the Batman R package^32^. Individual metabolite intensities were compared according to sex groups using Metaboanalyst 3.0 online tool^33^ for PCA, PLS-DA, volcano plot reconstruction and metabolite pathway enrichment analyses.

### Proteomic analysis

Nine livers from adult male and female medaka fish bred under unstressed control conditions were randomly pooled (one fish liver from each triplicate tank in each triplicate pool) and homogenized on ice with a Dounce homogenizer in 500 μL of a solution of 6 M guanidine hydrochloride, 500 mM triethylammonium bicarbonate buffer (TEAB, pH 8.3), 0.1% Triton X-100 and 10 μg of protease inhibitor mixture (Roche, Switzerland). The homogenates were centrifuged at 4 °C (12,000 g; 10 min), and then the supernatants were collected. Proteins were precipitated with cold acetone (−20 °C; overnight), centrifuged at 4 °C (2,000 g; 4 °C), and then resuspended in 500 mM TEAB with 6 M urea and 0.1% SDS. The protein concentration was measured using a micro-BCA kit (Sigma-Aldrich, USA), with BSA as a protein standard.

100 μg of each liver protein pool was used for digestion with 5 μg of proteomic-grade trypsin (Sigma-Aldrich, USA), reduced with 2 mM tris-(2-carboxyethyl)phosphine (TCEP) with and cysteine-blocked with 10 mM methyl methane-thiosulfonate (MMTS), prior to analyses with a Q Exactive™ Hybrid Quadrupole-Orbitrap™ mass spectrometer (Thermofisher Scientific). Liver protein digests were concentrated on C18 stages tips, recovered in 40 μL 2% aqueous TFA, 2% ACN before injection in triplicates (6 μL injected). NanoLC was performed on an Ultimate 3000 RSLCnano System (Thermofisher Scientific): digests were desalted on a trap column (Pepmap, C_18_ 300 μm x 5 mm, 5 μm 100 Å, Dionex) with water containing 2% ACN with 0.1% formic acid (solvent A) for 6 min, and the peptides were finally eluted from a separation column (Pepmap, C18 75 μm, x 500 mm, 3 μm 100 Å, Dionex). The separation gradient as optimized for the samples and is divided into 3 successive slopes: 2–20% in 120 min, 20–35% in 45 min and 35–80% ACN + 0.1% formic acid (solvent B) at a flow rate of 300 nL.min^−1^. Each MS spectrum acquisition (*m/z* 400–2000, 70,000 Res.) was followed by up to ten data dependent HCD MS/MS spectra (first fixed mass *m/z* 90, 17,500 Res., 30 normalized collision energy) with an isolation window *m/z* 2 and a dynamic exclusion window of 30 s.

All MS/MS-analyzed samples were analyzed using Mascot 2.4.1 (Matrix Science, UK) and X!Tandem with Scaffold software (version 4.5.1; Proteome Software, USA) to search Uniprot databases of Teleostei (downloaded in December 2015). The ion mass tolerance and the parent ion tolerance were set to 20 mDa and 10 ppm, respectively. The methyl methanethiosulfonate of cysteine was specified as fixed modifications. Oxidation of methionine and deamination of N and Q were specified as variable modifications. The Scaffold was used to probabilistically validate the protein identifications derived from MS/MS sequencing results. Normalized semi-quantifications and identification probability of male and female identified proteins were estimated using Scaffold+ default parameters from MS and MS/MS data for proteins presenting at least two peptides.

iTRAQ based quantitative proteomic was performed in triplicates for each experimental group on 3-pooled livers from nine livers from adult male and female medaka fish for each condition (CHT1, CHT2, CHT3 and Control) that were randomly pooled (one fish liver from each triplicate tank in each triplicate pool) as previously described^34^. Nano-LC-MS/MS analysis of the 8-plex tagged peptide digests was performed using the same top-10 strategy. A Quality Check (QC) sample was injected every 5 samples, in order to confirm that no significant drift of the analysis occurs, according to expected minimal identification number. Protein quantification was performed with Scaffold Q+ (4.5.1) using the isobaric tag peptide and protein identifications. Protein identifications were accepted if they could be established with more than 99.0% probability, and contained at least 2 identified peptides that were quantified using the centroid reporter ion peak intensity. Protein quantitative ratios were calculated as the median of all peptide ratios of the three consecutive runs. Quantitative ratios were log_2_ normalized for final quantitative testing, using control value set up as a reference sample in both sexes. Heatmap protein quantification was represented using Gene-E freeware (http://www.broadinstitute.org/cancer/software/GENE-E/) using Spearman correlation’s value for sample and protein clustering analyses.

### RNA-Seq

Two or three pooled livers from untreated adult males and females randomly selected from the three replicate tanks were homogenized using a bead beater. Total RNA of pooled female and male livers (6 biological replicates, for each sex) was isolated and purified using RNeasy Plus Mini Kit with gDNA eliminator spin (Qiagen). RNA quantity and quality were evaluated using Qubit RNA Assay Kit in Qubit^®^2.0 Fluorometer (Life Technologies, USA) and an Agilent Bioanalyzer 2100 eukaryote total RNA Pico series II chip (Agilent Technologies Inc., USA), respectively. The RIN values of all samples (3 pool samples for males and 4 pool samples for females) further selected for RNA-seq analysis were over 7.7. The transcriptome libraries were prepared from total RNA using Illumina TruSeq Stranded mRNA Sample Preparation kits (Illumina Inc., USA) following the manufacturer’s handbook. Briefly, the mRNA was purified using poly-T oligo-attached magnetic beads and then fragmented into small pieces that were then used for synthesizing the first- and second-strand cDNA. After end repair, single nucleotide A (adenine) addition and adaptor ligation, the fragments were amplified with a 10-cycles PCR program. The libraries were sequenced on the Illumina Hi-Seq1000 instrument using the 51 bp single-end sequencing strategy with the TruSeq SBS kit V3-HS 50-cycles (Illumina Inc., USA).

Raw reads were first cleaned by removing adaptors using Cutadapt-1.3 and only 51 bp-long reads were kept. The global quality of the reads was checked using the FastQC 0.10.1 and good global Phred scores (>30) were obtained in all the libraries. However, the analysis of the unicity of reads shows a high level of sequence duplications, so a step of duplicated reads removal was conducted using a python script that analyses the quality of reads and keeps the one with the best global quality score. Tophat2 (v2.0.10)^35^ was used to map the clean unique reads against the medaka genome (release 81) downloaded from Ensembl (ftp://ftp.ensembl.org/pub/release-81/fasta/oryzias_latipes/dna/). Multiple hits were removed by samtools (v0.1.18) and read counting on gene exons was accomplished by HTSeq-count (v0.6.1p1)^36^ in union mode against the annotation of medaka genomes downloaded from Ensembl (ftp://ftp.ensembl.org/pub/release-81/gtf/oryzias_latipes/). DESeq2 v1.8.1^37^ was used to do differential expressed gene analysis on the raw count data. Genes were considered differently expressed when the *p* value below 0.001, using the control group as reference. Furthermore, we only included genes with expression level of at least 4 |FC| in order to capture more physiologically relevant genes. Then, we identified 375 female- and 147 male-enriched transcripts (Supplementary Table S4). Messenger RNA expression levels were secondarily confirmed by RT-qPCR on 8 genes randomly selected within the expression intensity gradient that presented correlation coefficient greater than 0.95 between these two techniques in both sexes.

### Molecular network analysis

Molecular pathway was determined for our merged transcriptome and metabolome data using the Ingenuity Pathway analysis software (V01-04; Qiagen) with the Human orthologous of medaka proteins available from Ensembl online platform (http://www.ensembl.org), according to specific Ingenuity Knowledge Database (Genes and Endogenous Chemicals), which is a repository of biological interactions and functional annotations. The fold change values (females *vs* males) and *p* values calculated according to the quantifications of all replicates for 2214 gene expressions (FDR < 0.05) and 245 metabolites (HMDB numbers) were imported into IPA, then “Core Analysis” was performed, with default setting on liver tissue and relaxed filters, including both direct and indirect relationship between our dataset and the reference annotations, in order to interpret data in the context of biological pathways, molecular functions and networks.

## Additional Information

**How to cite this article**: Qiao, Q. *et al*. Deep sexual dimorphism in adult medaka fish liver highlighted by multi-omic approach. *Sci. Rep.* **6**, 32459; doi: 10.1038/srep32459 (2016).

## Supplementary Material

Supplementary Information
